# The Good, the Bad, and the Ugly of Dendritic Cells during Prion Disease

**DOI:** 10.1155/2015/168574

**Published:** 2015-11-30

**Authors:** Neil Andrew Mabbott, Barry Matthew Bradford

**Affiliations:** The Roslin Institute and R(D)SVS, University of Edinburgh, Easter Bush, Midlothian EH25 9RG, UK

## Abstract

Prions are a unique group of proteinaceous pathogens which cause neurodegenerative disease and can be transmitted by a variety of exposure routes. After peripheral exposure, the accumulation and replication of prions within secondary lymphoid organs are obligatory for their efficient spread from the periphery to the brain where they ultimately cause neurodegeneration and death. Mononuclear phagocytes (MNP) are a heterogeneous population of dendritic cells (DC) and macrophages. These cells are abundant throughout the body and display a diverse range of roles based on their anatomical locations. For example, some MNP are strategically situated to provide a first line of defence against pathogens by phagocytosing and destroying them. Conventional DC are potent antigen presenting cells and migrate via the lymphatics to the draining lymphoid tissue where they present the antigens to lymphocytes. The diverse roles of MNP are also reflected in various ways in which they interact with prions and in doing so impact on disease pathogenesis. Indeed, some studies suggest that prions exploit conventional DC to infect the host. Here we review our current understanding of the influence of MNP in the pathogenesis of the acquired prion diseases with particular emphasis on the role of conventional DC.

## 1. Introduction

**Figure 1:**
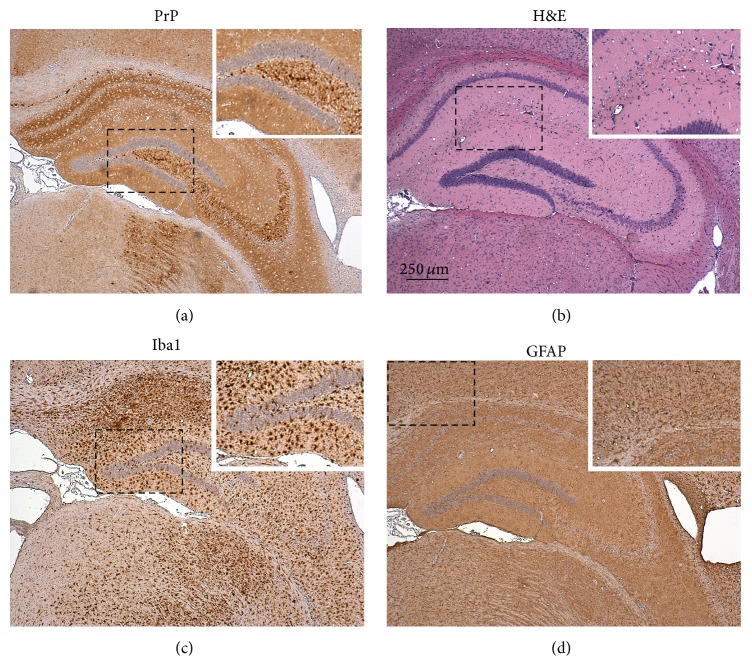
Neuropathological characteristics of prion disease within the brains of clinically affected mice. (a) Prion diseases are characterized by the presence of aggregations of abnormally folded, disease-specific prion protein (PrP) in affected tissues (brown). In the brain, as shown here, these accumulations are accompanied by extensive neuronal loss, spongiform change (indicated by vacuolation in panel “(b)”), reactive microglia (Iba1^+^ cells, panel “(c),” brown), and reactive astrocytes expressing high levels of glial fibrillary acidic protein (GFAP, panel “(d),” brown). Sections are counterstained with haematoxylin (blue).

**Figure 2:**
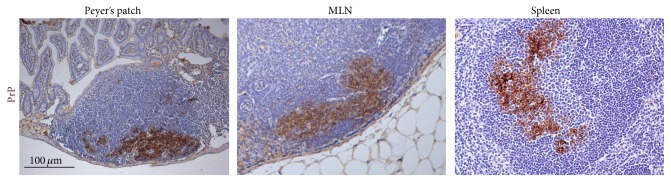
Stromal-derived follicular dendritic cells are important sites of prion accumulation and replication in the B cell follicles of secondary lymphoid tissues. Detection of high levels of abnormally folded, disease-specific prion protein (PrP, brown) in Peyer's patches, mesenteric lymph nodes (MLN), and spleen of a mouse infected with ME7 scrapie prions. Sections are counterstained with haematoxylin (blue).

Prion diseases, or transmissible spongiform encephalopathies, are subacute neurodegenerative diseases affecting humans and certain domestic and free-ranging animal species. These diseases are characterized by the presence of aggregations of PrP^Sc^, abnormally folded isoforms of the cellular prion protein (PrP^C^), in affected tissues. Although the precise nature of the infectious prion is still the subject of intense debate, prion infectivity copurifies with PrP^Sc^ which is considered to constitute the major component of the infectious agent [1, 2]. The accumulation of PrP^Sc^ in the central nervous system (CNS) of prion-infected hosts is accompanied by neuronal loss, spongiosis, and reactive glial responses (Figure 1). Some prion diseases appear to have idiopathic aetiology. These may arise spontaneously within the CNS (such as sporadic Creutzfeldt-Jakob disease (CJD)) or are associated with polymorphisms within the* PRNP* gene (which encodes PrP^C^) which some consider predisposes the prion protein to abnormally fold into the disease-specific isoform (such as Gerstmann-Straussler-Scheinker syndrome). Many other prion diseases, including natural sheep scrapie, bovine spongiform encephalopathy, and chronic wasting disease in cervids and kuru and variant Creutzfeldt-Jakob disease (vCJD) in humans, are acquired following exposure to prions, for example, by oral consumption of prion-contaminated food. For the efficient transmission of prions to the CNS after peripheral exposure (a process termed* neuroinvasion*), the replication of prions within secondary lymphoid tissues is crucial [3]. Within lymphoid tissues, prions replicate upon stromal-derived follicular dendritic cells (FDC) located within the B cell follicles [4–6] (Figure 2). Following their replication and amplification upon FDC, the prions subsequently spread along neurones of the sympathetic and parasympathetic nervous systems, accessing the CNS wherein they ultimately cause neurodegeneration resulting in the death of the host [7–10].

**Figure 3:**
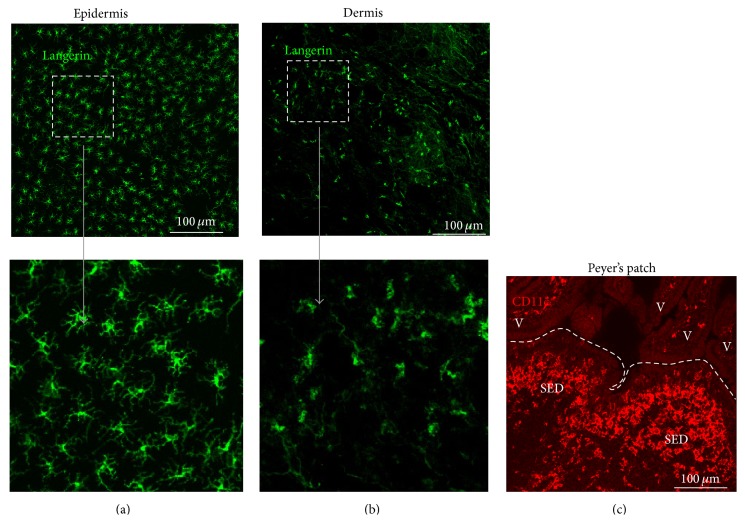
Mononuclear phagocytes (MNP) are a heterogeneous population of monocytes, macrophages, and dendritic cells and are abundant throughout the body. MNP are strategically situated at exposure sites such as in the epidermis or dermis of the skin (panels “(a)” and “(b),” resp.) and in the intestinal lamina propria where they provide a first line of defence against pathogens. (a and b) Whole-mount immunohistochemical detection of langerin^+^ Langerhans cells in the epidermis (green, panels “(a)”) and langerin^+^ conventional DC in the dermis (green, panels “(b)”). The boxed region in the upper panels is shown at higher magnification in the adjacent lower panels. (c) CD11c^+^ MNP (red) are abundant in Peyer's patches and the intestinal lamina propria. SED, subepithelial dome region on Peyer's patch; V, villus; broken line indicates the boundary of the epithelium overlying Peyer's patch.

Mononuclear phagocytes (MNP) arise from haematopoietic precursor cells within the bone marrow and are a heterogeneous population of monocytes, macrophages, and dendritic cells [11–13]. These cells are abundant throughout the body and possess a diverse range of functions based on the anatomic locations they occupy. For example, some MNP are strategically situated at exposure sites such as the skin or intestinal lamina propria to provide a first line of defence against pathogens by phagocytosing and destroying them in their phagolysosomal compartments. Others, such as conventional dendritic cells (DC), are potent antigen presenting cells and provide an important link between the innate and adaptive immune systems. These MNP are located to efficiently sample host tissues and fluids for pathogens and their antigens (Figure 3). The immature conventional DC at these sites are highly phagocytic. Following the uptake of pathogens or antigens, these cells typically undergo maturation and migrate via the lymphatics to the draining (regional) lymphoid tissue, such as the mesenteric lymph nodes (MLN) associated with the intestine [14], where they present the antigens to lymphocytes to initiate an antigen-specific (adaptive) immune response or induce tolerance [15]. Other MNP populations appear to play an important role within lymphoid tissues in the transfer of intact antigens to B cells [16, 17]. In this review, it is important to remember that the migratory, bone marrow-derived conventional DC [15] are entirely distinct from the stromal derived FDC [18, 19] which have been shown to be the critical sites of prion replication in lymphoid tissues [6]. The FDC, in contrast, are localized within B cell follicles of lymphoid tissues, derive from ubiquitous perivascular precursor cells [19], are tissue fixed and nonphagocytic. In contrast to conventional DC, FDC are long-lived cells which can retain native antigens on their surfaces for long periods.

Viable commensal bacteria can be recovered from DC migrating from the intestine [20] and some pathogenic microorganisms may exploit DC as an efficient way to infect host tissues [21]. In the transient absence of CD11c^+^ DC at the time of peripheral exposure, the early accumulation of prions in the draining lymphoid tissue was blocked and disease susceptibility reduced [22–24]. These data imply that prions may also exploit conventional DC to infect the host. Thus, in this review we discuss our current understanding of the role of MNP in the pathogenesis of the acquired prion diseases with particular emphasis on conventional DC.

## 2. Conventional DC: A Multifunctional Cellular Component of the Innate Immune System

**Figure 4:**
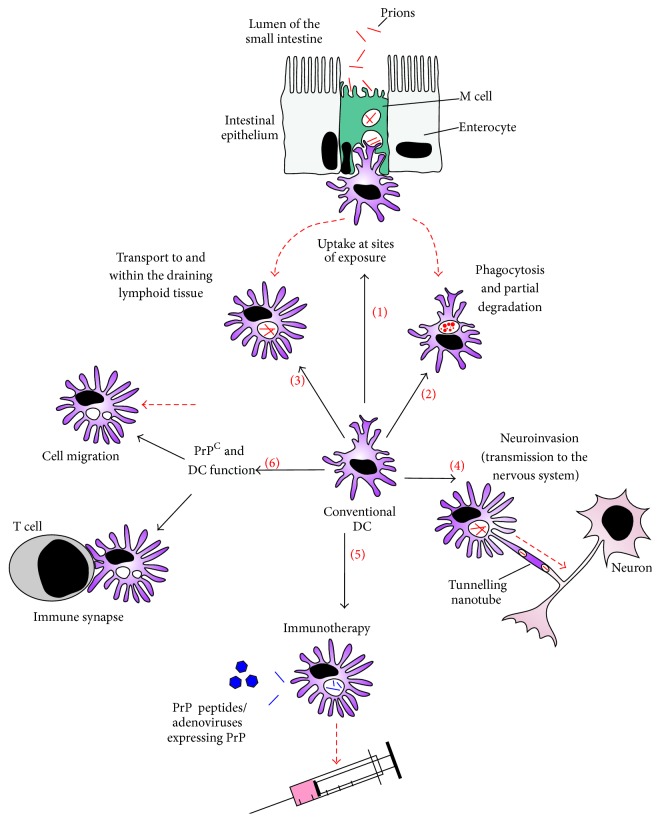
The influence of DC on prion disease pathogenesis. (1) Conventional DC are strategically placed throughout the mammalian host and are amongst the first cell populations to interact with prions. Following their uptake of prions DC have been proposed to exert a diverse range of contrasting effects on prion disease pathogenesis which may have a significant outcome on the spread of infection to the CNS. (2) Some studies have suggested that DC may help to protect the host against infection by attempting to sequester and destroy the prions [25–29]. (3) Others suggest that prions may exploit the migratory characteristics of DC to facilitate their efficient propagation from the site of exposure to the lymphoid tissues [22–24, 34, 39]. (4) DC may also play an important role in the subsequent transfer of prions to the CNS by bridging the gap between the immune and peripheral nervous systems [36, 61, 91–93]. (5) The adoptive transfer of PrP peptide-loaded DC into mice can overcome host tolerance towards PrP and prolong survival time after prion infection. This implies that DC could be manipulated to provide immunotherapeutic protection against prion diseases [54, 106, 107]. (6) The physiological function of cellular PrP^C^ is uncertain but in DC may play a role in the immune synapse or in the regulation of cell migration [51, 52].

MNP such as conventional DC exhibit a diverse array of functions in the mammalian innate immune system. This is reflected in the various ways in which they may interact with prions, and, by doing so, impact on prion disease pathogenesis (Figure 4). Following their uptake by conventional DC, the prions may (i) activate innate immune responses and be sequestered and partially degraded within the cell's phagolysosomal compartments; (ii) undergo amplification (replication) since these cells express the substrate PrP^C^; (iii) activate acquired immune responses and induce a specific immune response to prions as conventional DC are potent antigen-presenting cells; (iv) be conveyed from the site of exposure to sites of prion replication within the draining lymphoid tissue. The sections below describe the many studies which have attempted to address the potential contribution of these roles to prion disease pathogenesis.

## 3. *In Vitro* Cultivated DC Can Acquire and Destroy Prions

“Immature” conventional DC are highly phagocytic cells and have the potential to sequester and destroy prions in a similar manner to that in which they process peptide antigens for presentation to T cells in association with MHC class II. Data from several independent studies support this hypothesis and have shown that* in vitro *cultivated DC-like cells can readily acquire and degrade prions [25–28]. Within these cells, the prion-specific PrP^Sc^ appears to be preferentially degraded by cysteine proteases [29]. These data are congruent with data from similar studies using macrophages which show they can also acquire and degrade prions after extended* in vitro* exposure [30, 31]. Whether these data accurately reflect the handling and processing of prions by conventional DC* in vivo* is uncertain since these cells can retain high levels of infectious prions in infected rodents [32–36]. Furthermore, when macrophages are depleted* in vivo* in prion-infected hosts, higher concentrations of prions are recovered from their lymphoid tissues [37, 38]. In contrast, depletion of CD11c^+^ cells impedes the early accumulation of prions in the draining lymphoid tissue [22–24, 39] (see below).

## 4. DC Are Not Important Sites of Prion Replication

Although conventional DC are typically considered to internalize antigens which they then process into short peptides and present them on their surfaces to T cells, some MNP populations including certain conventional DC subsets appear to be equipped with both degradative and nondegradative antigen handling pathways [40, 41]. These distinct pathways may enable conventional DC to present processed peptide antigens to T cells or native antigens to B cells. During prion infection DC can sequester high levels of prions [32–36], but these cells are highly unlikely to be acting as important early sites of prion replication or amplification. Expression of the cellular prion protein, PrP^C^, is obligatory for prion replication, and MNP including conventional DC in mice, humans, and cattle express PrP^C^ on their surfaces [42–44]. However, several studies have shown that prion replication within the secondary lymphoid tissues and disease pathogenesis are not influenced by the absence of PrP^C^ expression in haematopoietic cells [6, 45–48]. Thus, the role of DC during prion pathogenesis is more complex than simply acting as sites of prion replication.

## 5. The Enigmatic Function of PrP^ C^ in the Immune System

The cellular prion protein, PrP^C^, is 30–35 kDa glycoprotein linked to the cell surface via a glycosylphosphatidylinositol anchor. The precise function of PrP^C^ in mammalian cells remains elusive, but the expression of PrP^C^ by many immune cell populations, including conventional DC, implies a role in immune function [42–44]. However, mice that lack PrP^C^ expression in the haematopoietic compartment display no obvious immune deficit and are able to maintain antigen-specific antibody responses and affinity maturation [49]. Some studies have suggested that PrP^C^ may regulate phagocytosis. Upon further scrutiny, a separate study revealed that the reduced ability of MNP to phagocytose apoptotic cells in* Prnp* ^−/−^ mice was due to effects on a linked locus encoding the signal regulatory protein *α* (*Sirpa*) gene rather than the absence of PrP^C^ expression [50]. Microscopical analyses show PrP^C^ accumulates at contact sites between T cells and antigen-loaded conventional DC implying a role in the immune synapse between these cell populations. Consistent with this, the absence of PrP^C^ in antigen-presenting cells impacted on their ability to stimulate T cells [51].

A separate study has proposed that PrP^C^ may regulate human monocyte migration by modulating cell adhesion dynamics [52]. The authors propose that PrP^C^ regulates *β*1-integrin-mediated adhesion by modulating the remodelling of the actin cytoskeleton through the RhoA-cofilin pathway.

## 6. Induction of Specific Immunity against Prions

Although conventional DC are potent antigen-presenting cells and play an important role in the induction of antigen-specific immune responses, these cells are unlikely to play a role in the induction of specific immunity against prions. The prion protein is tolerated by the host immune system due to the widespread expression of PrP^C^ throughout the body. This prevents the induction of specific cell-mediated and antibody-mediated immune responses to PrP^Sc^, the major component of infectious prions [53]. Despite this, a cell-based immunotherapy approach may be possible against prions as experiments have shown that the adoptive transfer of PrP peptide-loaded conventional DC into mice can overcome host tolerance towards PrP and prolong survival time after peripheral prion exposure [54].

## 7. DC and the Propagation of Prions to Draining Lymphoid Tissues

Some DC populations have been shown to have the ability to capture and retain unprocessed antigens in their native states and transfer them intactly to naïve B cells to initiate a specific antibody response [17]. Viable commensal bacteria can also be recovered from conventional DC migrating from the intestine [20]. The demonstration that some pathogenic microorganisms appear to exploit migratory DC to enable their delivery to lymphoid tissues [21, 55–57] raised the hypothesis that DC may play a similar role in the initial delivery of prions from the site of infection (such as the gut lumen) to the draining lymphoid tissues (such as the gut-associated lymphoid tissues after oral exposure). This hypothesis was further supported by the observation that some migrating intestinal DC in the afferent mesenteric lymph had acquired PrP^Sc^ following its injection into the gut lumen [34]. Subsequent studies have since shown that, in the absence of migratory DC at the time of peripheral exposure, the early accumulation of prions in the draining lymphoid tissue and the subsequent spread of disease to the CNS are both impeded [22–24, 39]. However, not all DC subsets appear to share this property. For example, whereas the depletion of CD11c^+^ cells (using CD11c-DTR-eGFP-tg mice) dramatically impedes oral prion pathogenesis [22], specific depletion of CD8^+^CD11c^+^ cells (using CD11c-N17Rac1-tg mice) does not [24]. Similarly, prion pathogenesis following infection via skin lesions was impaired in the specific absence of CD11c^+^ langerin^−^ dermal DC but was not affected in the absence of epidermal Langerhans cells or langerin^+^ dermal DC [39].

Chemokines help to attract lymphocytes and DC to lymphoid tissues and control their positioning within them. For example, the chemokines CCL19 and CCL21 are constitutively expressed by stromal cells within the T cell zones and mediate the homing of chemokine receptor CCR7-expressing naïve T cells and mature DC towards them [58]. The positioning of DC within the interfollicular T cell regions of Peyer's patches and their steady-state migration from Peyer's patches to the MLN are likewise dependent upon CCR7-CCL19/CCL21-signalling [59]. However, the CCL19/CCL21-mediated attraction of DC is unlikely to influence prion neuroinvasion from Peyer's patches since oral prion pathogenesis is unaffected in* plt* mice which lack CCL19 and CCL21 [33]. This observation is consistent with data from other studies showing that Peyer's patches in the small intestine, not the MLN which collect the lymph and cells draining the intestine [14], are the critical sites of prion accumulation and neuroinvasion after oral prion exposure [3, 60]. Prion pathogenesis is likewise unaffected in the specific absence of T cells [61].

The demonstration that the accumulation of prions upon FDC in the draining lymphoid tissues was prevented in the absence of DC at the time of exposure [22–24, 39] implied that prions exploit these cells to access the draining lymphoid tissue, perhaps by using them as “Trojan horses.” The detection of PrP^Sc^-containing DC within the villous lacteals and submucosal lymphatics in the intestines of sheep soon after exposure to prions by oral infection or by injection into ligated gut loops implies a similar role [62–64]. Distinct DC subsets have been described that can transport native antigen to B cells* in vivo* [17, 65, 66]. The chemokine CXCL13 is highly expressed by FDC and follicular stromal cells in the B cell follicles of lymphoid tissues and modulates the homing of CXCR5-expressing B cells into them [67, 68]. The migration of certain populations of splenic DC and dermal DC into B cells follicles is also mediated by CXCL13-CXCR5 signalling [69, 70]. During virus infection, DC within the medullary sinus have been shown to capture lymph-borne influenza virus particles and subsequently migrate to the FDC-containing B cell follicles [71]. Further studies are clearly necessary to determine whether, after acquiring prions, DC similarly migrate similarly towards B cell follicles and in doing so infect FDC.

Although several studies suggest that DC may play an important role in the initial delivery of prions to and within the draining lymphoid tissues, the possibility that some of the prions may enter these tissues in a cell-free manner should not be excluded [39, 72, 73].

## 8. How Do DC Acquire Prions?

Whether DC acquire and endocytose prions via a specific receptor or receptors is uncertain, but the neurotoxic prion protein fragment PrP_106–126_ is a chemoattractant for monocyte-derived DC [74]. Some MNP subsets express cellular PrP^C^ highly which may itself act as a receptor for prion-specific PrP^Sc^ [42–44]. However, if DC do acquire some prions in a PrP^C^-dependent manner, it does not play a major role in disease pathogenesis. The propagation of prions from various peripheral sites of exposure to FDC and their subsequent neuroinvasion are not influenced by a lack of PrP^C^ expression by haematopoietic cells [6, 45–48]. These observations suggest the existence of other receptors on the surfaces of DC besides PrP^C^ that they may use to acquire prions.

The FDC within the B cell follicles are considered to acquire prions in the form of complement-opsonized complexes [75, 76]. Conventional DC may similarly indirectly acquire prions following their opsonisation by complement components such as C1q and C3 [72, 77]. The complement C1q-dependent uptake of prions by conventional DC appeared to be complement receptor- (CR-) mediated [77]. The identity of the specific receptor which mediates the uptake of complement-opsonized prions by conventional DC is uncertain, but many candidate molecules such as CR1 (CD35), CR2 (CD21), CR4 (CD11c/CD18), calreticulin, CD93, and SIGN-R1 (CD209b) are expressed by specific populations of these cells and can bind C1q [72, 77]. In other studies, it is interesting to note that the SIGN-R1-mediated uptake of influenza virus by DC lining the medullary sinus of lymph nodes stimulates their subsequent migration towards FDC [71]. After oral exposure, it is possible that the prions are acquired from the gut lumen in complex with dietary ferritin [78]. Finally, since MNP such as conventional DC are highly phagocytic, they may simply acquire prions nonspecifically as the cells constitutively sample their microenvironment, for example, via micropinocytosis.

## 9. Plasmacytoid DC also Sequester Prions

The plasmacytoid DC are a distinct subset of MNP which rapidly secrete large amounts of type I interferon (IFN-*α*/*β*) in response to foreign nucleic acids such as during virus infection [79]. One study has shown that plasmacytoid DC, like conventional DC, can also sequester high levels of infectious prions during infection [36]. The consequences that this may have on prion disease pathogenesis are uncertain. Plasmacytoid DC are unlikely to play a role in the propagation of prions to the draining lymphoid tissues since these cells do not migrate in the lymphatics during the steady-state or following activation [80]. Plasmacytoid DC also express negligible levels of PrP^C^, even after activation, so like classical DC they are unlikely to be important sources of prion replication [81]. Prion disease also does not induce the synthesis of significant levels of IFN [82–84], and treatment of mice soon after prion infection with polyriboinosinic-polyribocytidylic acid (poly(I:C)), which stimulates type I IFN production, does not alter disease pathogenesis [85, 86]. Splenic plasmacytoid DC may simply be attempting to sequester and destroy prions following their amplification by FDC. However, some studies have suggested that plasmacytoid DC and classical DC [36, 87] may play a role in prion neuroinvasion by facilitating the subsequent propagation of prions to peripheral nerves (see below).

## 10. DC and the Propagation of Prions between the Immune and Nervous Systems

Following their amplification upon FDC prions subsequently infect the peripheral nerves within the lymphoid tissue [8, 9, 88]. The prions then spread along the nerves of both the sympathetic and parasympathetic nervous systems and subsequently infect the CNS where they cause neurodegeneration leading to the death of the host [9, 10]. How prions spread between FDC and peripheral nerves is not known as these cells do not make significant physical contacts or synapses. Within peripheral tissues, there is much crosstalk between MNP and peripheral nerves. For example, in the intestine MNP/conventional DC are abundant in the muscular layer where they interact with enteric neurones and help regulate gastrointestinal motility [89, 90]. Given their migratory properties, experiments have sought to determine whether DC might also bridge the gap between FDC and peripheral nerves during prion disease.

Data from* in vitro* coculture studies show that prion-infected DC could potentially transfer prions to primary neurones or mouse neuroblastoma N2a cells [91–93]. Efficient prion transfer between these populations required cell-cell contact [92, 93]. Furthermore, when fixed bone marrow-derived DC were used, this activity was blocked implying an active process was required [92]. Data from a detailed* in vitro* study have proposed that tunnelling nanotubes (TNT), thin membrane-bound cylinders of cytoplasm which can connect neighbouring cells, might represent a novel method through which the intracellular exchange of prions between these cells may occur [91]. Within the TNT the PrP^Sc^ appears to travel in endolysosomal vesicles [94]. Whether significant transfer of prions between cells by TNT occurs in the dynamic environment of the lymphoid tissues* in vivo* remains to be determined. However, the analysis of lymphoid tissues from HIV patients shows intercellular transfer via a similar mechanism is possible. Xu and colleagues revealed that HIV-1-infected macrophages were able to establish long range intercellular connections (consistent with TNT) with B cells [95]. These intercellular conduits were exploited by the virus to deliver a virus encoded immunosuppressive factor to B cells to enable it to suppress the humoral response.

Prions have also been proposed to be released from infected cells in the form of small endosomal-derived vesicles termed exosomes [96]. Therefore, during prion infection DC may also release significant amounts of infectious prions in this manner and in doing so enhance their ability to infect neighbouring cells [36].

An* in vivo* study has suggested that prion-infected DC alone are potentially sufficient to transfer infection directly to the nervous system. Immunodeficient* Rag1* ^−/−^ mice lack T and B cells and are indirectly deficient in FDC. As a consequence these mice are refractory to peripheral infection with prions [61]. Despite this, live prion-infected DC were sufficient to transmit disease after intravenous injection into* Rag1* ^−/−^ mice [32]. Since these mice lack mature FDC and are unable to replicate prions in their lymphoid tissues, these data implied that the DC had transferred the prions directly to the peripheral nervous system. However, an independent study using FDC-deficient* Tnfr1* ^−/−^ mice was unable to demonstrate significant direct infection of the nervous system by prion-infected DC [35]. The precise reason for the discrepancy between these two studies is uncertain but may relate to the much higher density of peripheral nerves in the spleens of* Rag1* ^−/−^ mice when compared to* Tnfr1* ^−/−^ mice [35]. Clearly further studies are necessary to determine the precise contribution of DC and DC-derived tunnelling nanotubes or exosomes to the transfer of prions between FDC and peripheral nerves* in vivo*.

A subset of MNP with apparent conventional DC characteristics has been described in the mouse brain [97]. Within the brains of variant CJD patients deposits of PrP^Sc^ have been described in vascular-associated DC [98], and another study has proposed that CD205^+^ (DEC-205) expressing DC may also migrate into the murine brain during prion disease [99]. Under certain circumstances, monocytes may also traffic to the brain and, in doing so, act as potential vectors for the delivery of pathogens such as virus or prions or misfolded aggregates of Alzheimer's disease-related amyloid *β* protein [100]. The possibility cannot therefore be entirely exclude that prion-infected conventional DC contribute to the establishment of prion infection in the CNS. Complement components C1q and C3 associate with PrP^Sc^ in the brains of prion-infected mice [101], raising the possibility that prions are acquired by DC in the CNS in a complement-dependent manner [72, 77].

The detection of prion-specific PrP within the circumventricular organs of the brain has been reported to be an early feature in scrapie-affected sheep [102]. Due to the presence of their fenestrated capillaries, the circumventricular organs are important sites of molecular exchange between the blood stream and the CNS. However, during prion disease monocytic infiltration into the circumventricular organs is not observed arguing against the cell-associated haematogenous spread of prions into the CNS. Studies in mice also show that CCR2-deficiency and absence of recruitment of circulating monocytes do not significantly impact on prion disease pathogenesis within the CNS [103]. The depletion of sympathetic nerves dramatically impedes the spread of prions from lymphoid tissues to the CNS [10]. Conversely, prion pathogenesis after peripheral exposure is exacerbated by treatments which increase the density of sympathetic nerves in lymphoid tissues [10] or in mice in which the distance between FDC and sympathetic nerves is reduced [88]. These findings are consistent with the conclusion that prions initially infect the CNS via their spread along peripheral nerves rather than direct haematogenous transfer.

## 11. Conclusions

### 11.1. The Many Faces of DC during Prion Disease

As described above, DC have been proposed to exert a diverse range of contrasting effects on prion disease pathogenesis which may have a significant outcome on the spread of infection to the CNS. Some studies have suggested that DC may help to protect the host against infection by attempting to sequester and destroy the prions. Others suggest that prions may exploit the migratory characteristics of prions to facilitate their efficient propagation from the site of exposure to the lymphoid tissues. DC may also play an important role in the subsequent transfer of prions to the CNS by bridging the gap between the immune and peripheral nervous systems (Figure 4).

### 11.2. DC or Not DC?

**Figure 5:**
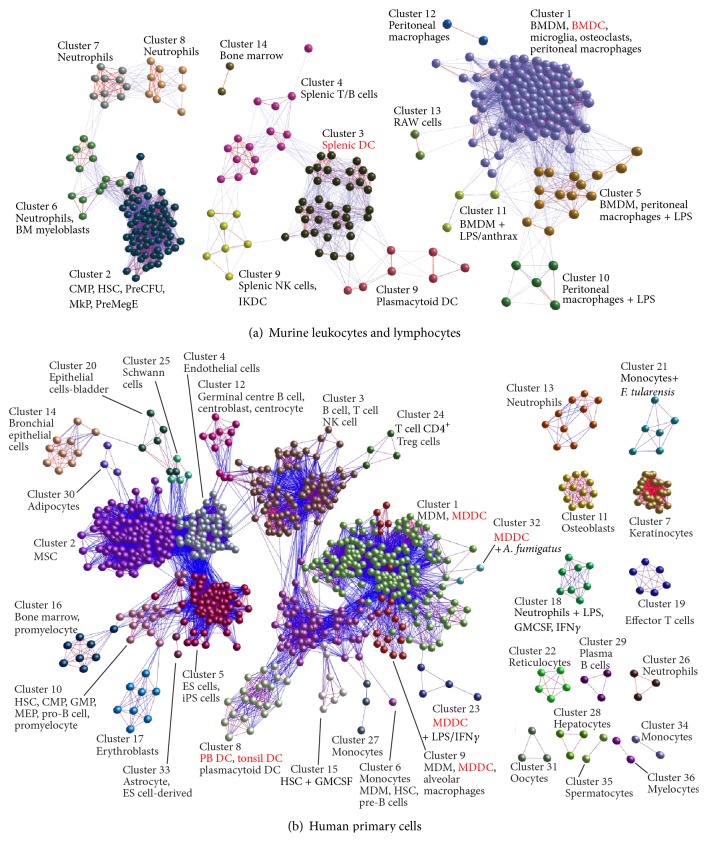
Transcriptional analyses show that mouse bone marrow-derived DC and human monocyte-derived DC are indistinguishable from macrophages. Clustering of cell subsets based upon their global gene expression profiles. In panels “(a)” and “(b)” Pearson correlation matrices were prepared by comparing the microarray data sets derived from all samples used in each study ([11] and [105], resp.). Graphs were then constructed using only those sample-to-sample relationships greater than *r* = 0.9 and clustered with an MCL inflation value of 2.2. Each node represents an individual microarray data set and the edges are coloured on a sliding scale according to the strength of the correlation (red, *r* = 1.0; blue, *r* = 0.9). Each cluster of samples was assigned a different colour. Each of these analyses shows that at the transcriptomic level the* in vitro* bone marrow-derived DC (BMDC) or monocyte-derived DC (MDDC) prepared from mice (panel “(a)”) and humans (panel “(b)”) are indistinguishable from macrophages and do not cluster with conventional DC enriched from tissues such as the spleen, tonsils, or peripheral blood. The tissue DC, BMDC, and MDDC data sets are highlighted in red font. Reference [11] provides full details about the sources of all the 304 individual microarray data sets used in “(a).” Reference [105] provides full details about the sources of all the 745 microarray data sets used in “(b).” BMDC, bone marrow-derived dendritic cells; BMDM, bone marrow-derived macrophage; anthrax,* Bacillus anthracis* edema toxin; CMP, common myeloid progenitors; ES, embryonic stem cell; GMP, granulocyte monocyte progenitors; HSC, haematopoietic stem cell; IKDC, interferon-producing killer DC; MDDC, monocyte-derived DC; MDM, monocyte-derived macrophage; MEP, megakaryocyte-erythroid progenitor cell; MkP, megakaryocyte progenitors; MSC, mesenchymal stem cells; NK, natural killer; PreCFU-E, preerythroid progenitors; PreMegE, premegakaryocyte/erythroid; PB, peripheral blood; Treg, regulatory T cell. Panel “(a)” is reproduced from [11] with permission from Elsevier. Panel “(b)” is reproduced from [105] under the terms of the Creative Commons Attribution License 2.0.

While it is evident from data described above that the actions of certain MNP populations may significantly influence the outcome of a peripheral prion infection, it is uncertain whether the cells involved actually are DC. Indeed there is much controversy over whether DC and macrophages can be separated based on either their functions or transcriptomes [13, 104]. Most of the studies in experimental mice have defined conventional DC based on their expression of a limited number of cell surface markers such as the integrin CD11c (integrin alpha x (Itgax)). Murine conventional DC do express CD11c highly, but this integrin is not restricted to these cells as most MNP express CD11c, including the majority of the MNP within the intestine [12]. Large numbers of DC-like cells can be prepared* in vitro* following the treatment of bone marrow cells or monocytes with GM-CSF and IL-4. The cells obtained from these preparations do share many typical characteristics of conventional DC, such as expression of high levels of CD11c and potent antigen-presenting activity, but macrophages can also share these characteristics [13, 104]. Furthermore, at the transcriptomic level these* in vitro* bone marrow-derived or monocyte-derived DC prepared from mice and humans are indistinguishable from macrophages and do not cluster with conventional DC enriched from tissues [11, 13, 105] (Figure 5). While data clearly show that MNP may have many important effects during prion infection, further studies are necessary to distinguish between the separate roles of DC and macrophages in disease pathogenesis in experimental rodents and natural host species.

### 11.3. DC-Based Antiprion Immunotherapy

As well as playing an important role in the establishment of a peripherally acquired prion infection, current data suggest that conventional DC may potentially be manipulated in order to provide immunotherapeutic protection against peripherally acquired prion diseases. As proof-of-principal studies have shown, a conventional DC-based immunotherapy approach can overcome host tolerance towards PrP^C^ and impede peripheral prion disease pathogenesis [54, 106, 107]. Finally, the early accumulation of prions in the draining lymphoid tissue is impeded and disease susceptibility reduced in the absence of CD11c^+^ cells at the time of exposure [22–24, 39]. Therefore, the identification of the molecular factors which influence the handling of prions by CD11c^+^ MNP may reveal novel targets for therapeutic intervention in the initial phase of infection with these invariably fatal neurodegenerative diseases.

## Abbreviations

CJD: Creutzfeldt-Jakob disease
CNS: Central nervous system
CR: Complement receptor
DC: Dendritic cells
FDC: Follicular dendritic cells
IFN: Interferon
MLN: Mesenteric lymph nodes
MNP: Mononuclear phagocytes
PrP: Prion protein
TNT: Tunnelling nanotubes.
